# Emulating synaptic response in *n*- and *p*-channel MoS_2_ transistors by utilizing charge trapping dynamics

**DOI:** 10.1038/s41598-020-68793-7

**Published:** 2020-07-22

**Authors:** Shubhadeep Bhattacharjee, Rient Wigchering, Hugh G. Manning, John. J. Boland, Paul K. Hurley

**Affiliations:** 1 Tyndall National Institute, and the School of Chemistry, University College Cork, Cork, Ireland; 2 Advanced Materials and Bioengineering Research (AMBER) Centre and School of Chemistry, Trinity College Dublin, Dublin 2, Ireland; 3 Department of Physics and Astronomy, University of Manchester, Manchester, M13 9PL UK

**Keywords:** Electrical and electronic engineering, Nanoscale devices

## Abstract

Brain-inspired, neuromorphic computing aims to address the growing computational complexity and power consumption in modern von-Neumann architectures. Progress in this area has been hindered due to the lack of hardware elements that can mimic neuronal/synaptic behavior which form the fundamental building blocks for spiking neural networks (SNNs). In this work, we leverage the short/long term memory effects due to the electron trapping events in an atomically thin channel transistor that mimic the exchange of neurotransmitters and emulate a synaptic response. Re-doped (*n*-type) and Nb-doped (*p*-type) molybdenum di-sulfide (MoS_2_) field-effect transistors are examined using pulsed-gate measurements, which identify the time scales of electron trapping/de-trapping. The devices demonstrate promising trends for short/long term plasticity in the order of ms/minutes, respectively. Interestingly, pulse paired facilitation (PPF), which quantifies the short-term plasticity, reveal time constants (τ_1_ = 27.4 ms, τ_2_ = 725 ms) that closely match those from a biological synapse. Potentiation and depression measurements describe the ability of the synaptic device to traverse several analog states, where at least 50 conductance values are accessed using consecutive pulses of equal height and width. Finally, we demonstrate devices, which can emulate a well-known learning rule, spike time-dependent plasticity (STDP) which codifies the temporal sequence of pre- and post-synaptic neuronal firing into corresponding synaptic weights. These synaptic devices present significant advantages over iontronic counterparts and are envisioned to create new directions in the development of hardware for neuromorphic computing.

## Introduction

Despite the enormous success of scaling the complementary metal–oxide–semiconductor (CMOS) transistor, the inability to control the rising computational complexity and associated power consumption have revealed inherent limitations of the von-Neumann architecture^1^. In the von-Neumann model, the logic and memory elements are functionally and physically separated, and connected via a data bus which is expected to facilitate rapid and efficient exchange of information^2^. With the increasing operational frequencies and computing requirements, one of the primary bottlenecks in terms of data throughput and power consumption/heat dissipation is centered on the capability of these data exchange buses^3^. The recognition of this limitation was concurrent with the development of artificial intelligence (AI) algorithms that mimic the low-power learning algorithms in the biological brain^4,5^. The biological computing paradigm is essentially non-Von Neumann; computation and memory are tightly interwoven in functionality and morphology^5^. In contrast to the sequential instruction set in CMOS architecture; learning and computing in the brain happen in a parallel, event-based manner. Therefore, the attempt to integrate an essentially non-Von Neumann AI algorithm into a von-Neumann CMOS architecture was generally unsuccessful due to two core issues vis-à-vis complexity/scalability and power consumption^6–8^. To replicate the analog computing scheme of one synapse, at least 10 CMOS transistors are warranted^9^. Considering that these architectures demand tightly interwoven elements, the scalability of these systems becomes a primary challenge^8^. Furthermore, in conjunction with the aforementioned issue, large power (> 1,000 W) is consumed by such platforms to perform tasks that the human brain achieves with < 4 W^10^. This points to the immediate need to explore electronic devices that can mimic the synaptic/neuronal response and are compatible with the CMOS fabrication process.

Memristive devices have been employed to successfully demonstrate synaptic behavior and emulate learning rules with a variety of physical mechanisms (resistive switching, phase change, spintronic etc.)^6,11^. This approach presents the dual advantages of scalability and very low power consumption^12^. However, because these devices are two-terminal, the signal transmission is inhibited during the learning operation, where the output signal is fed back to the synaptic device^13^. Since the signal transmission and learning function cannot be carried out simultaneously, the emulation of a natural synaptic response is hindered, and the complexity of the read-out/learning circuitry is significantly increased. To address this issue, three-terminal ‘iontronic’ transistors were developed where both signal transmission (through source-drain terminals) and learning (through the gate terminal) can be achieved simultaneously^14,15^. While the use of ‘movable’ alkali metal ions through a gel electrolyte intuitively matches the signal transmission mechanism in a biological synapse; however, this approach will be difficult to implement in the alkali metal–ion free policy of CMOS fabrication facilities^14^.

**Figure 1:**
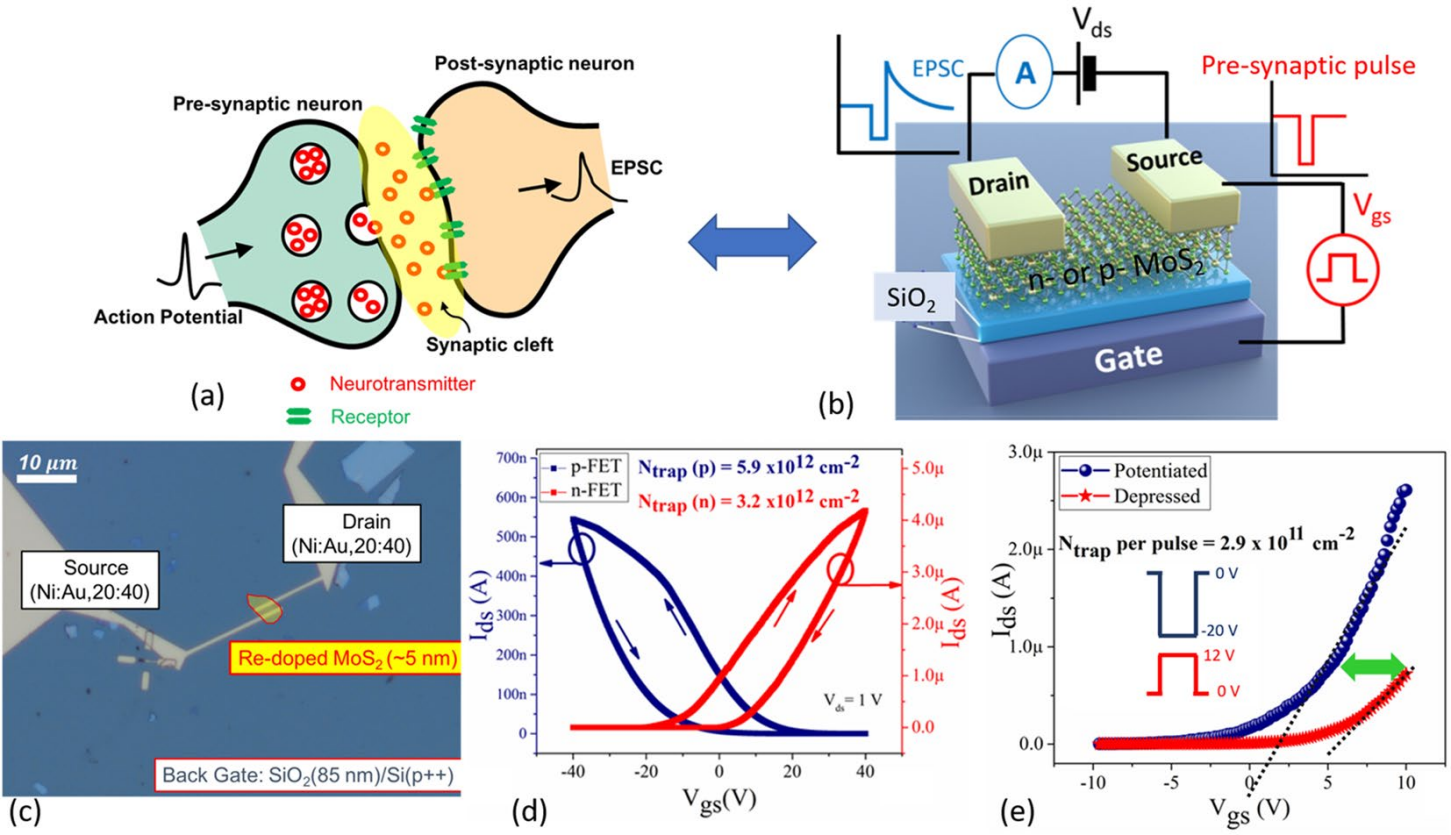
(**a**) Schematic representation of the synaptic response where action potentials control the release and absorption of neurotransmitters, with an associated excitatory postsynaptic current (EPSC), (**b**) The measurement scheme is illustrated in the cartoon, where charge trapping and de-trapping dynamics in an atomically thin channel 2D material (in our case Re- or Nb- doped MoS_2_) is used to mimic the synaptic response, where the presynaptic pulse is applied on the back-gate terminal and the excitatory post-synaptic current [EPSC] is measured at the source-drain terminal at a constant drain bias of 1 V. (**c**) Optical microscope image of a fabricated Re-doped MoS_2_ transistor on an SiO_2_ (85 nm) substrate which also serves as the back-gate. (**d**) DC characterization of a hysteresis loop for both Re- (*n*-type, red curve) and Nb- (*p*-type, blue curve) doped MoS_2_ transistors showing clear evidence of electron trapping and de-trapping. (**e**) Shift in threshold voltage after 50 consecutive pulses to the gate, in order to determine the average charge trapping/de-trapping after each pulse.

In this work, we demonstrate that the dynamics of charge trapping and de-trapping in a single *n*- and *p*-channel MoS_2_ transistor can be effectively employed to mimic a synaptic response (Fig. 1a,b). Owing to their inherent ‘thinness’, electronic transport in two-dimensional materials demonstrates an enhanced susceptibility to the influence of trapped charges in the surrounding medium^16–18^. This is generally observed by a reduced mobility and a large hysteresis in the device transfer characteristics^19^. The presence of a hysteresis, where the drain current becomes a multi-value variable with respect to the gate voltage is a serious issue for digital computing. However, as we shall demonstrate in this work the same hysteresis induced multi-states can be effectively engineered to mimic synaptic memory and learning. First, we examine the trapping and de-trapping dynamics of electron traps using pulsed measurements, assess their stability and the ability to tune many intermediate conductance states. Next, we perform a careful evaluation of the essential synaptic behaviors such as potentiation and depression (P-D), pulse paired facilitation (PPF) and spike time-dependent plasticity (STDP), which codify the ‘event-based’, parallel, analog-computing paradigm in these devices. Remarkably, the time constants for short and long-term phase in these devices are commensurate with those observed in biological synapses. We envision that the controlled engineering of a simple physical mechanism (trapping dynamics in 2D semiconductor/insulator material systems) can help re-shape the development of synaptic elements for next-generation neuromorphic computing.

## Results and discussion

MoS_2_ flakes of Rhenium (Re) *n*-doped and Niobium (Nb) *p*-doped serve as the channel material for *n*-FET and* p*-FET, respectively, on thermally grown SiO_2_/p++ Si (Fig. 1c) (Please see “Methods” for more details). The study was conducted for transistors with both polarities to emphasize the universality of the physical mechanisms and serve as a platform for future CMOS integration. The choice of a constrained channel thickness enabled through the use of an atomically thin 2D semiconductor, is instrumental in maximizing the influence of charge trapping and de-trapping events in the gate oxide on the measured drain current and hence the synaptic weights. Electrical measurements were performed in two configurations. First, standard DC measurements using a B1500 semiconductor device analyzer (SDA) were performed with the underlying SiO_2_ used as the back gate. Second, the pulsed measurements were performed using the Keithley 4200 semiconductor characterization system (SCS) with additional pulse measurement units (PMU). The pulsed signal was applied to the back gate and the response of the drain current (I_ds_) to these pulses was measured at a constant drain-source bias (V_ds_) (Fig. 1b) (please see “Methods” for more details). All measurements were performed in ambient conditions. In this case, the drain current serves as the excitatory postsynaptic current (EPSC), which is a direct measure of the device conductance as a function of time. The pre- and post-synaptic pulses, which are applied on the SiO_2_/Si++, back gate and the MoS_2_ channel can be thought of as an analog to the synaptic fluid where neurotransmitters (in this case trapped charges) are released and absorbed. In (Fig. 1d) we examine the DC characteristics of the devices, where the clockwise and anti-clockwise hysteresis for the *n*-FET and *p*-FET, respectively. This can be attributed to the trapping and de-trapping of electrons from donor-like traps in the MoS_2_/SiO_2_ structure, as commonly seen in other thin-film semiconductors^17,20^. In back-gated MoS_2_ transistors, the primary source of trapped charges has been attributed to the MoS_2_/SiO_2_ interface and surface absorbents/water dipoles on the top MoS_2_ surface^21^. The hysteresis behavior can be understood as follows: sweeping of the gate to positive biases increases the occupancy of electron traps, thus inducing a positive (negative) shift of the threshold voltage of the* n*-FET (*p*-FET) and hence decreasing (increasing) the channel conductance for the same gate bias. The equivalent trap density (N_trap_) at the MoS_2_/SiO_2_ interface can be estimated by the following expression,

1 $${\text{N}}_{{{\text{trap}}}} = {\text{ C}}_{{{\text{ox}}}} \left| {{\text{V}}_{{{\text{th}}}} \left( {{\text{for}}} \right) \, - {\text{ V}}_{{{\text{th}}}} \left( {{\text{rev}}} \right)} \right|/{\text{q,}}$$

where C_ox_ = 4 × 10^–8^ F/cm^−2^ is the oxide capacitance of the SiO_2_ back gate, V_th_ is the threshold voltage of the (for) forward and (rev) reverse sweep and q is the elemental charge constant. The N_trap_ of 3.2 × 10^12^ cm^−2^ for *n*-FET and 5.9 × 10^12^ cm^−2^ for *p*-FET is estimated for a V_bg_ swing of ± 40 V. A larger trap density and hence more defective interface of Nb-MoS_2_ (with SiO_2_) as compared Re-MoS_2_ is further evidenced by the degraded sub-threshold slope of the *p*-FET vis-à-vis the *n*-FET (please see Supporting Information S1 for semi-log plot of transfer characteristics). The field-effect mobility of the *n*-FET ~ 26.2 cm^−2^ V^−1^ s^−1^ and *p*-FET ~ 12.6 cm^−2^ V^−1^ s^−1^ can also be evaluated using the standard long channel approximation,

2 $$\mu_{FE} = \frac{L}{{C_{ox} W V_{ds} }}\frac{{dI_{ds} }}{{dV_{gs} }},$$

where L/W is the length to width ratio of the transistor and V_gs_ is the gate-source voltage, at a source-drain voltage (V_ds_) of 50 mV. The field-effect mobility values calculated for these devices are smaller than those with an un-doped channel, which is expected due to the additional impurity scattering^18^. To gain further insight into the charge trapping behavior, the hysteresis of the transistors was measured as a function of sweeping the V_gs_ sweep range and delay/sweep time (Supporting Information S2). The magnitude of the hysteresis loops, and the associated charge trapping, is determined by the maximum V_bg_ and the hold time in accumulation. Next, we proceed to the focus of this work; using controlled pulses to trap and de-trap electrons from the MoS_2_ channel and hence modulate the channel conductance, which represents the EPSC. As illustrated in (Fig. 1e), for a *n*-channel device a large change in the threshold voltage is observed before (red curve) and after (blue curve) the application of a series of 50 pulses to the back-gate with a pulse height of − 20 V and width of 500 ms (indicated by blue potentiation pulses in the figure inset). The average density of trapped charges per pulse can be calculated using Eq. (1) to be ~ 2.9 × 10^11^ cm^−2^. Furthermore, the initial position of the threshold voltage can be recovered by applying the same number of pulses of the opposite polarity + 12 V (indicated by red depression pulses). This suggests the ability to control different conductance states of the device repeatedly and reversibly using appropriate pulses applied to the back gate. A more nuanced examination of the same effect will be demonstrated shortly in the form of potentiation and depression measurements. The same effect is also reproduced for the *p*-channel device but with the opposite polarity for potentiation and depression (please refer Supporting Information S3).

**Figure 2:**
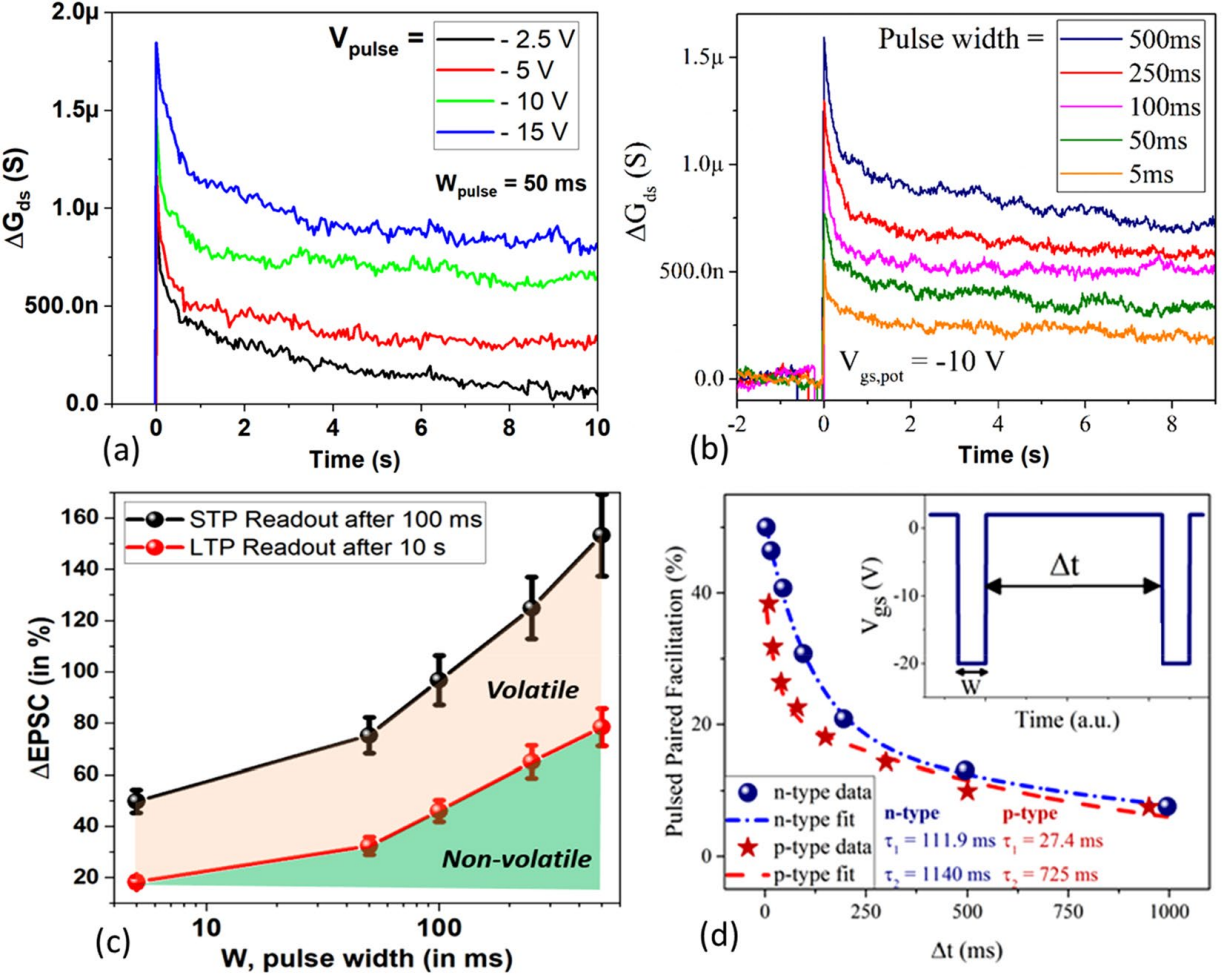
Pulsed measurements: Transient response of EPSC conductance as a function of (**a**) pulse height and (**b**) pulse width with a single pulse applied at time, t = 0 s for a *n*-channel MoS_2_ FET, at a constant V_ds_ = 1 V, baseline/rest value of V_bg_ =  + 2 V (**c**) Delineation of short- and long-term plasticity effects seen in the charge trapping devices, which are responsible for motor functions and experience-based learning in synapses. (**d**) Quantification of short-term plasticity using pulse paired facilitation measurements which shows time constants closely resembling those from a biological synapse for both* n*- and *p*-type devices. Please note V_ds_ = 1 V and 2 V for *n*- and *p*-type devices respectively.

The foundational basis of the biological brain to learn, memorize and forget hinges on the plasticity of synapses i.e. the ability to change the ‘conductance state’ depending on the previous history of action potentials^22^. The plasticity of the brain can be broadly sub-divided into long and short-term plasticity (LTP, STP), distinguished by their characteristic retention time(s). While the LTP is postulated to be responsible for experience-dependent modification of the synaptic weights (i.e. learning), the STP is largely effective for motor functions, auditory and visual perception etc^23^. Therefore, we focus on the STP and LTP behavior of the charge trapping devices by examining the transient response of the EPSC (or channel conductance) when a single pulse is applied at time, t = 0. Figure 2a,b illustrates the response of the channel conductance (EPSC) as a function of time when pulses of different height and width are applied to the back gate of the *n*-channel MoS_2_ transistor. The back-gate pulse polarity is selected to drive the *n*-channel MoS_2_ MOSFET to the off condition. The plot shows the evolution of the EPSC after the pulse has returned to the baseline value of 2 V, which demonstrates a sharp rise from the pre-pulse level, followed by a rapid decrease and subsequent slower decrease, indicative of the two time constants associated with the trapping/emission of electrons from defect sites. The two time constants associated with the electron trapping process is more clearly seen in the log time plot (see Supporting Information S4) which has been observed in back gated MoS_2_ MOSFETs^24^. The fast (~ 0.2 s) and slow (~ 5 s) time constants for *n*- and *p*-type device are extracted using a double exponential function for a pulse height and width 10 V and 100 ms respectively (see Supporting Information S5). The time constants for three *n*-type and two *p*-type devices are presented in Supporting Table S1. The presence of two-time constants for electron trapping or de-trapping is central to the emulation of a synaptic response. As we shall demonstrate shortly, using a pulsed paired facilitation (PPF) technique, the initial fast transient (typically < 1 s after the gate voltage pulse is applied) can accurately codify the short-term plasticity^25,26^. The change in the conductance of the synaptic transistor with respect to the baseline and the decay rate can be modulated by varying the pulse height and width respectively. As seen in Fig. 2a,b**,** for a pre-synaptic pulse width of 50 ms, which is in the same range of action potentials as in biological systems, the conductance value shows a distinguishable ‘memory effect’ with reasonable stability for the measurement window of > 10 s. Furthermore, the long-term retention of these devices can be extended to a few minutes by modulating the number of trapped charges introduced at the MoS_2_/dielectric interface, which demonstrates reasonable stability of conductance state with a very slow decay (see Supporting Information S6). This behavior can be taken to represent long-term stability. Eventually, there is a very gradual decay of the memory effect due to electron capture by defect sites. However, this phenomenon can be controlled in future design iterations by possibly including a trapping and tunneling layer as commonly implemented in modern floating-gate memories. Figure 2c delineates the short/long-term plasticity into volatile and non-volatile memory effects as a function of pulse width^27^.

As indicated earlier, we perform pulse-paired facilitation (PPF) measurements to extract the characteristic time constants for short-term plasticity^26^. In PPF measurements we monitor the EPSC when the device is subjected to a pair of pre-synaptic pulses (of the same magnitude) separated by a short time duration, Δt, typically in the order of 10–1,000 ms^26^. As shown in Supporting Information S7, the EPSC amplitude generated by the second pulse is greater than the first when a pulse of W = 5 ms is separated by a duration, Δt = 75 ms. The PPF index can be extracted by the formula

3 $$PPF = \frac{{\Delta EPSC_{Peak} }}{{\Delta EPSC_{1} }} \times 100\% ,$$

where the ΔEPSC_Peak_ is the difference between the peak current amplitude between the second and first pulse and ΔEPSC_1_ is the difference between the peak after the first pulse and the baseline (before the first pulse was applied)^27^. Figure 2d illustrates the PPF% as a function of pulse separation Δt from 10 to 1,000 ms for both *p*- and *n*-type devices. As is observed for biological synapses, the PPF% rapidly falls with increasing pulse separation. This can be explained as follows, let us suppose when the first pre-synaptic pulse is applied electrons are emitted from *n* trap sites in the device (neurotransmitters in a biological synapse) which is seen with the EPSC current value reaching a peak value, say I_1_ (*n*) which is a function of *n*. After a short time Δt, which is much smaller than the time required to reach steady-state conductance, a few trapped sites re-capture electrons, making the number of trapped sites *n-Δn*. When the second pre-synaptic pulse is applied, let us assume (for the sake of simplistic understanding) that electrons are emitted from *n* trap sites, taking the total tally of charge carriers to 2*n*-*Δn* and therefore changing the peak instantaneous current to I_2_(2*n-Δn*) such that I (2*n*) > I_2_(2*n − Δn*) > I_1_(n). Since the re-capture of electrons into trap-sites is an exponential function of time *Δn ∝ e* ^Δt^; therefore, for Δt → 0, I_2_ → I (2*n*) (PPF% ≤ 50%) and Δt → ∞, I_2_ → I_1_ (PPF% = 0). The PPF% exhibited by our samples can be fit to a double exponential function,

4 $$PPF_{fit} = A_{1} e^{{ - \frac{\Delta t}{{\tau_{1} }}}} + A_{2} e^{{ - \frac{\Delta t}{{\tau_{2} }}}}$$

where τ_1_ and τ_2_ are the relaxation time constants associated with fast- and slow- term relaxation phases. The extracted values of (τ_1_, τ_2_) for the *n*-FET and *p*-FET are (111.9 ms, 1,140 ms) and (27.4 ms, 725 ms). By coincidence, these values are strikingly close to those of the biological synapse (40 ms, 300 ms)^28^.

**Figure 3:**
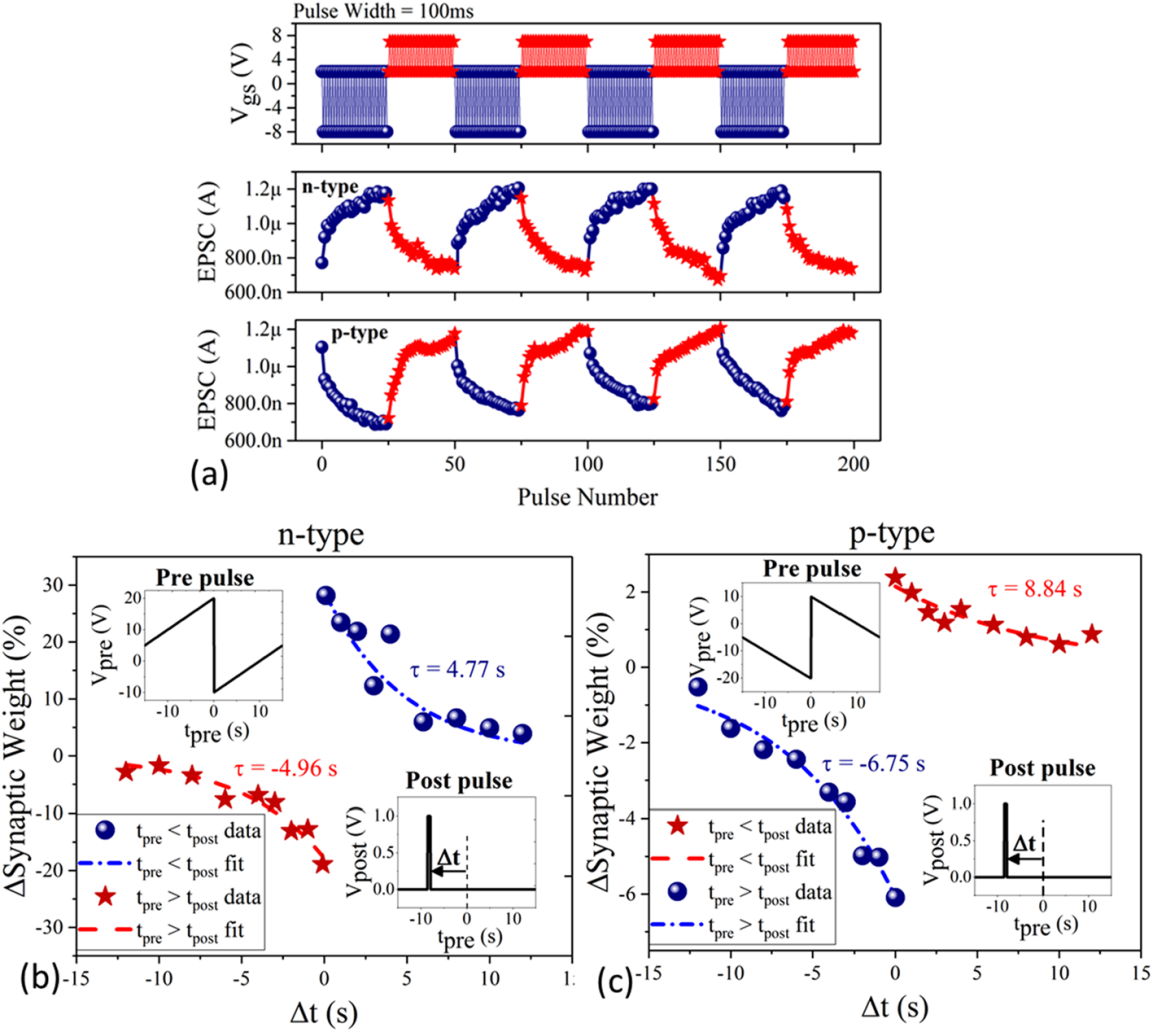
(**a**) Potentiation and depression measurements show the multi-cycle ability to traverse through at least 50 analog conductance states for both *n*- and *p*-type devices. Please note V_ds_ = 1 V and 2 V for *n*- and *p*-type devices respectively. Spike time-dependent plasticity that encodes the temporal firing of pre-synaptic neurons as channel conductance (ΔG%), synaptic weight (%) (please see Supporting Information S9 for more details) shows excellent resemblance to biological synapse for both (**b**) *n*-type and (**c**) *p*-type devices.

Next, we proceed to examine potentiation-depression (P-D) and spike time-dependent plasticity (STDP) in these synaptic devices, which is a crucial property in regard to the realization of neuromorphic circuits. P-D refers to the ability of the synaptic device to cyclically traverse through a multitude of analog conductance states when subjected, ideally, to pulses of consistent heights and widths^29^. Figure 3a demonstrates that both the *n*-FET and *p*-FET devices can access up to 50 such conductance states in a repeatable manner over several cycles. The pulse height (− 10 V, + 5 V, with a baseline of 2 V) and width (100 ms) was optimized to ensure multi-cycle behavior without any significant shift to the initial baseline between consecutive cycles. The conductance states were determined by measuring the EPSC in the 200 ms rest period and with V_bg_ (rest) = 2 V in between two consecutive pulses at a constant V_ds_ of 1 V and 2 V for the *n*- and *p*-type transistors respectively. The higher value of V_ds_ for the *p*-type transistor was to compensate for the lower mobility in Nb-MoS_2_. The repeatable, multi-cycle potentiation-depression characteristics of one such device for 600 pulses (12 cycles) is presented in Supporting Information S8.

Here, it shall be instructive to estimate the energy dissipation per pulse during potentiation and depression for this device. The average energy consumed per pulse E = ${\Delta }I_{ds} V_{ds} t_{pulse}$ where, ${\Delta }I_{ds}$ is average change in drain source current per potentiation/depression pulse V_ds_ = 1 V and $t_{pulse}$ = 100 ms. Using the data presented in Fig. 3a we arrive at 1.6 (2.8) nJ for n(p)-FET. We compare this with the one of the best values reported from three-terminal devices with similar architecture but utilizing ion-transport^30^. In this work, Yang et.al. report a value of ~ 25 pJ for a 100 ms pulse width, which was achieved at a much lower source drain voltage of 50 mV. Since our devices show near-linear output characteristics $I_{ds} \propto V_{ds}$ ,the energy consumption scales as square of drain voltage $E \propto V_{ds}^{2}$. Therefore, in principle, at a drain voltage of 50 mV one can project our devices to work at 4(7) pJ per pulse for n(p)-FET demonstrating, as expected, a lower energy dissipation per pulse for an electron device compared to an iontronic device. In another report, Zu et al.^31^ utilize nanowire structures (and hence very small W/L ratio in a transistor) to achieve very small I_ds_ (in pA) for small V_ds_ (20 mV) to demonstrate ~ fJ power consumption, the lowest in literature. Though these reports may be scientifically interesting, the arbitrarily low drain voltage and current biasing conditions used in these reports will be challenging to implement in a practical circuit without low-noise amplifiers at each stage. Nonetheless, it is instructive to calculate the lowest theoretical limit of power consumption in the charge-trapping synaptic devices reported in this work. Since the fundamental mechanism of our device depends on the trapping and de-trapping of charge carriers the lowest possible limit of energy consumption will be E = ${\Delta }Q_{trap} V_{ds}$, where ${\Delta }Q_{trap}$ is the number of traps contributing per pulse. This of course assumes that all other DC, parasitic and transistor biasing currents to be zero and that a generation of ${\Delta }Q_{trap}$ at the MoS_2_/SiO_2_ interface induces equal number of charges in the channel. As demonstrated in Fig. 1e and Supporting Information Fig S3, one could calculate the ${\Delta }N_{trap}$ (trap density) per pulse by measuring the shift in the threshold voltage of the transistor averaged over many pulses. Subsequently ${\Delta }Q_{trap}$ = $q {\Delta }N_{trap} A$, where q is the fundamental charge constant, 1.6 × 10^–19^ and A is the area of the device. Considering our devices which are ~ 5 μm^2^ in area, this yields a value of 0.64 fJ (for 50 ms pulse) and 2.56 fJ (500 ms pulse) for a V_ds_ of 1 V.

In addition to systematic traversing of conductance states, a synaptic device is also expected to emulate commonly known learning rules, whereby the channel conductance is encoded as a function of the temporal firing of the pre- and post- synaptic neurons. Amongst many postulated rules, STDP is widely believed to be the basis of learning, information storage, and refinement of neuronal pathways, as popularized by the adage ‘neurons that fire together, wire together’^32^. In STDP, the conductance of the channel (*ΔG*) is potentiated or depressed based on whether the pre-synaptic neuron fires before or after the post-synaptic neuron and the magnitude of the potentiation or depression is exponentially determined by the time difference (Δt) between the two firings^31^. Mathematically, this can be expressed as:

5 $$For \Delta t \ge 0, \Delta G \propto e^{{ - \frac{{\left| {\Delta t} \right|}}{{\tau_{ + } }}}} ;\quad For \Delta t \le 0, \Delta G \propto - e^{{ - \frac{{\left| {\Delta t} \right|}}{{\tau_{ - } }}}} ,$$

where $\Delta t \ge 0$ ($\Delta t \le 0$) refers to the case where pre-synapse fires before (after) post synapse and τ_+_, τ_−_ are the respective time constants. To examine STDP behavior in our devices we use a commonly prescribed mapping function for three-terminal geometries that transform/codifies the time difference between two pulses to varying magnitudes of the pre-synaptic pulses^33,34^. This is enabled by using a 2 × 1 multiplexer connected to the gate terminal, where the post-synaptic neuron is used as a control signal to modulate the pre-synaptic pulse and grounded terminal (see Supporting Information S9 for pulsing scheme drain current characteristics)^35^. Figure 3b,c demonstrates the STDP response for *n*- and *p*-type FETs, both showing exponential modulation of channel conductance (%G) as a function of Δt, where,

6 $$\left( {G, \, synaptic \, weight \, \% } \right) \, = \, \left( {G_{before} - G_{after} } \right)/G_{before} \times 100\%$$

G_before_ and G_after_ are the conductance of the channel before and after the application of the post-synaptic pulse. The shape of the symmetric Hebbian post and pre-synaptic pulses are also illustrated in the insets. The extracted time constants for potentiation and depression (τ_+_, τ_−_) for *n*-FET and *p*-FET is (4.77 s, 4.96 s) and (8.84 s, 6.65 s) respectively.

## Conclusions

In summary, we demonstrate how the simple process of electron trapping/de-trapping events in a back-gated MoS_2_ transistor can electrically mimic the release and absorption of neurotransmitters/ions in a biological synapse. The use of charge trapping devices offers significant advantages in terms of reliability and manufacturability over “iontronic” devices. To generalize our observations and demonstrate complementary behavior, which shows promise for CMOS integration, we examine both *n*- (Re-doped) and *p*-doped (Nb-doped) MoS_2_ transistors as three-terminal synaptic devices. The charge trapping and release from defect states in the devices exhibits time constants which allows the emulation of both short- and long-term plasticity, an important property in biological synapses which aids learning and memory. Interestingly, PPF measurements on these devices, which quantify the short-term plasticity reveal time constants that closely resemble those from a biological synapse. Furthermore, potentiation-depression characteristics showing at least 50 analog conductance states and the ability of these devices to codify commonly used learning rules such as STDP lays special emphasis on the flexibility and adaptability of charge trapping devices. The extraction of the plasticity, PPF and STDP related time constants from this work can be used to initiate circuit level simulations for SNNs. The efficacy of trapping/de-trapping events in the gate oxide in emulating the behavior of biological synapses opens up new avenues in the design of neuromorphic devices.

## Methods

Re-doped and Nb- doped MoS_2_ crystals procured from HQ Graphene were exfoliated on 85 nm SiO_2_ using the scotch-tape method. Thin flakes in the optimal range of 4–8 nm were identified using optical contrast and confirmed using atomic force microscopy. Flakes thinner than this suffer from reduced drain currents and mobility because of a larger band-gap and poorer contacts; whereas, drain current in thicker films become less sensitive to the trapped charges in the MoS_2_/SiO_2_ system, and hence are undesirable. On the selected flakes, source-drain contacts with a channel length and width of 1 μm were defined using electron beam lithography (EBL). Next, a mid-gap metal Ni/Au (20/40) which shows reasonable contacts to both n and p doped MoS_2_ was deposited using electron beam evaporation, followed by a lift-off step. Figure 1c presents an optical image of one of the fabricated transistors. Electrical measurements were performed in two configurations. First, standard DC measurements using a B1500 semiconductor device analyzer (SDA) were performed with the underlying SiO_2_ used as the back gate. Second, the pulsed measurements were performed using the Keithley 4200 semiconductor characterization system (SCS) with additional pulse measurement units (PMU).

## Supplementary information

Supplementary information
