# Supplementary material for: Emulating synaptic response in n- and p-channel MoS2 transistors by utilizing charge trapping dynamics
Source: Sci Rep. 2020 Jul 22;10:12178. doi: 10.1038/s41598-020-68793-7 (PMC7376145; doi:10.1038/s41598-020-68793-7)
Supplement: Supplementary file 1 — Supplementary information [file 41598_2020_68793_MOESM1_ESM.pdf]

Supporting Information

Emulating Synaptic Response in *n*- and *p*- channel MoS_2 Transistors by Utilizing Charge Trapping Dynamics.

Shubhadeep Bhattacharjee*[1], Rient Wigchering,[1] Hugh G Manning,[2] and John. J. Boland,[2] Paul K. Hurley[1*]

[1] Tyndall National Institute, University College Cork, Cork, Ireland

[2] Advanced Materials and Bioengineering Research (AMBER) Centre, Trinity College Dublin, Dublin 2, Ireland

*Corresponding authors: s.bhattacharjee@tyndall.ie, paul.hurley@tyndall.ie.

Supplementary Information: Semi-log plot of transfer characteristics, hysteresis loops as a function of sweep voltage and delay time, trap per pulse in a *p*-type transistor, extraction of fast and slow time constants with device statistics, conductance stability for few minutes, EPSC measurement in PPF, 600 cycles of potentiation and depression measurements, STDP measurement schematic and pulsing scheme with raw data.

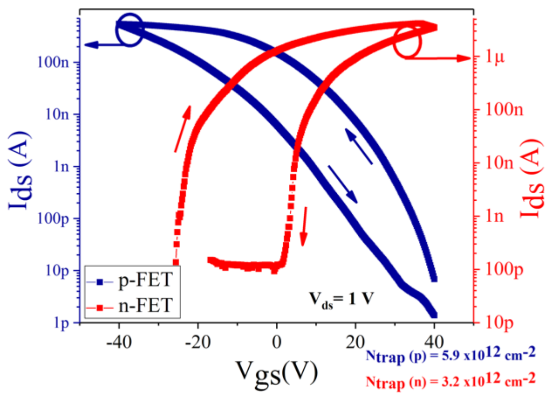

Fig S1. DC characteristics of *n*-FET (red) and *p*-FET (blue) in semi-log plot. The *p*-FET demonstrates a degraded sub-threshold swing in comparison to *n*-FET indicating more defective interface with SiO_2 as also evidenced by larger number of traps for the same gate sweep of ± 40 V.

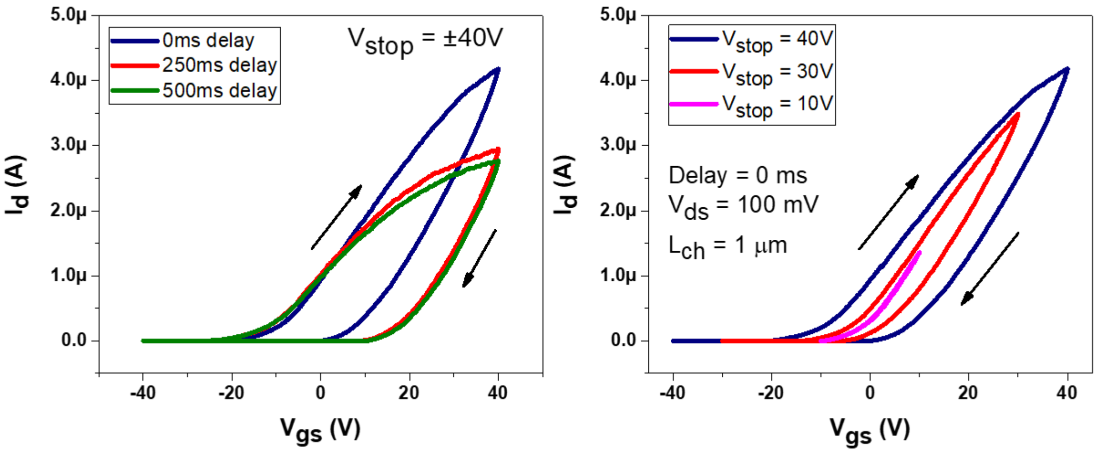

Fig S2. Broadening of the hysteresis loop with an increase in (a) delay time between two consecutive measurement points (b) sweep range of the gate voltage. The results show classical signatures of electron charge trapping for a *n*-FET.

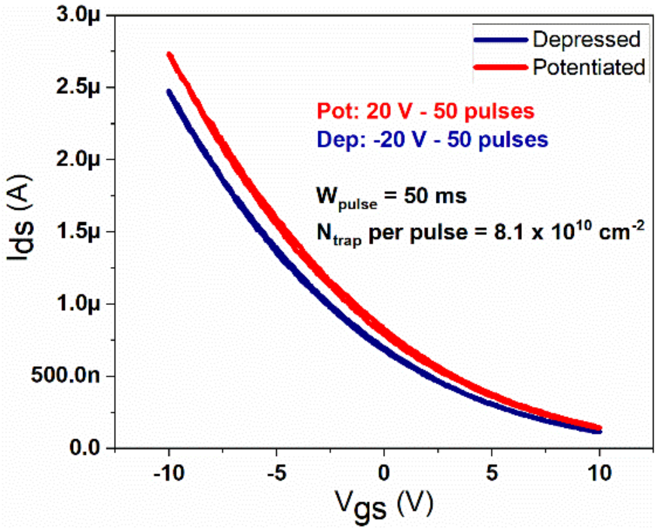

Fig S3: A *p*-type transistor subjected to 50 consecutive pulses of 50 ms for potentiation (+20 V) and depression (-20 V). The shift in the threshold voltage (at constant V_{ds}= 1 V) was used to calculate the N_{trap} per pulse ~ 8.1 $x10^{10}$ cm^{-2}

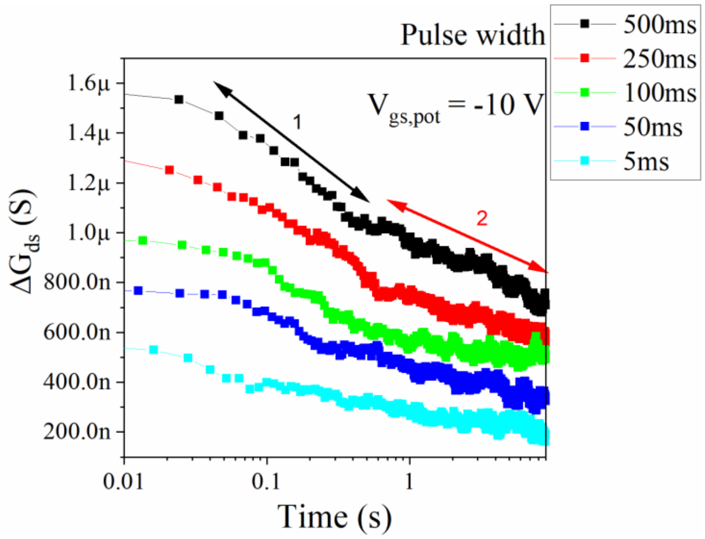

Fig S4: The Fig 1(b) from main manuscript re-plotted in log time scale to illustrate two distinct electron de-trapping relaxation regimes.

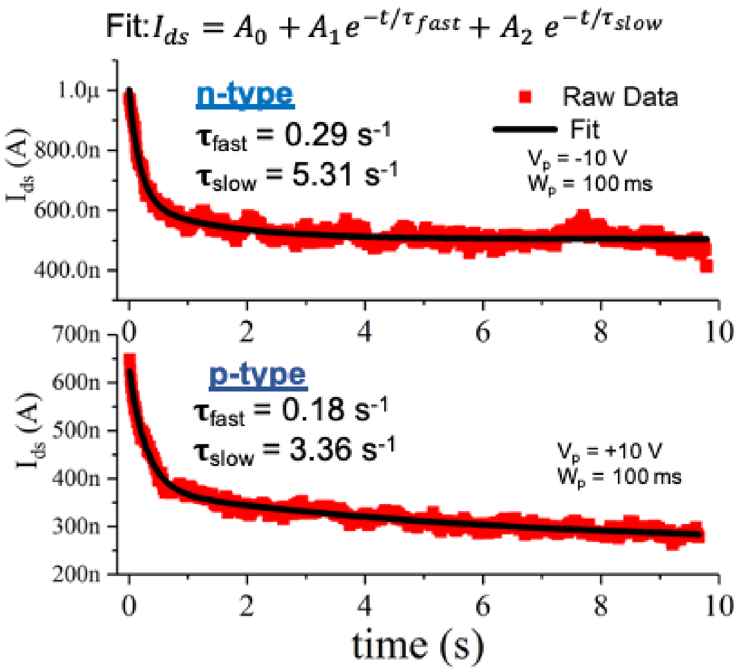

Fig S5: Fast and slow time constants extracted for the best *n*- and *p*- type device fitted to a double exponential. The devices having most distinct fast and slow time constants are good candidates to extract secondary synaptic parameters. Please note that these time constants are in response to a single pulse, unlike the case of the PPF measurements with are in response for a pair of pulses and are used to characterise the initial fast decay (short-term plasticity).

Device ID	doping	N_{trap}, (cm^{-2}) ±40 V V_{gs} sweep	Fast time constant (s)	Slow time constant (s)
**Dev 1**	**Re (n)**	**3.2 x10^{12}**	**0.29**	**5.31**
Dev 2	Re (n)	3.6 x10^{12}	0.23	2.26
Dev 3	Re (n)	2.9 x10^{12}	0.51	4.83

**Dev 4**	**Nb (p)**	**5.9 x10^{12}**	**0.18**	**3.36**
Dev 5	Nb (p)	4.8 x10^{12}	0.33	2.85

Table S1: The fast and slow time constants extracted for three *n*-type and two *p*-type devices.

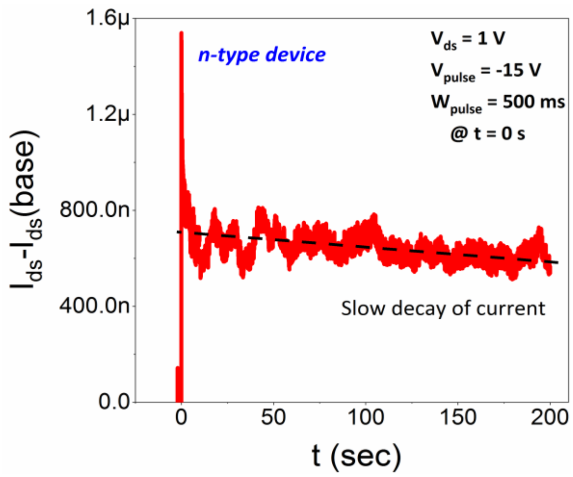

Fig S6: Slow decay and reasonable retention of synaptic conductance over > 3 mins for a pulse height of 15 V and pulse width of 500 ms.

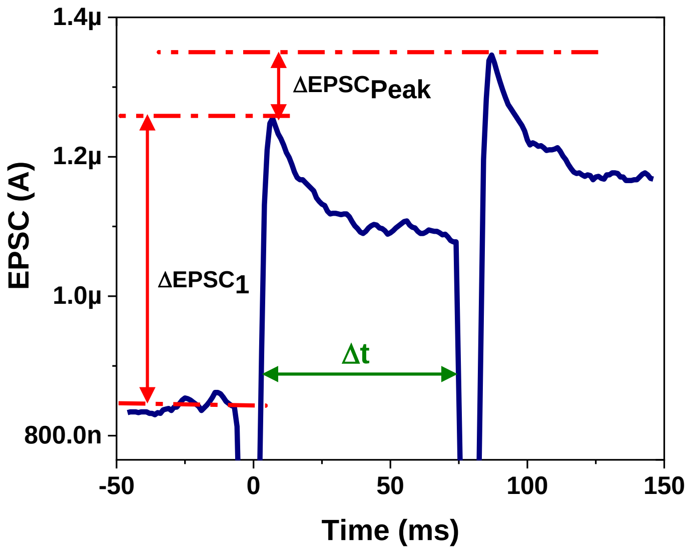

Fig S7: Pulse paired facilitation measurements: EPSC amplitude when two closely spaced pulses are applied to the gate terminal of an *n*-channel MoS_2 transistor, pulsing conditions: V_{bg} (pre-pulse/rest) = +2 V and V_{bg} (during pulse) = -20 V, pulse width, W = 5 ms,separated by a duration, Δt = 75 ms, measured at a constant V_{ds} = 1 V. The $\Delta EPSC_{Peak}$ is the difference between the peak current amplitude between the second and first pulse and $\Delta EPSC_1$ is the difference between the peak after the first pulse and the baseline.

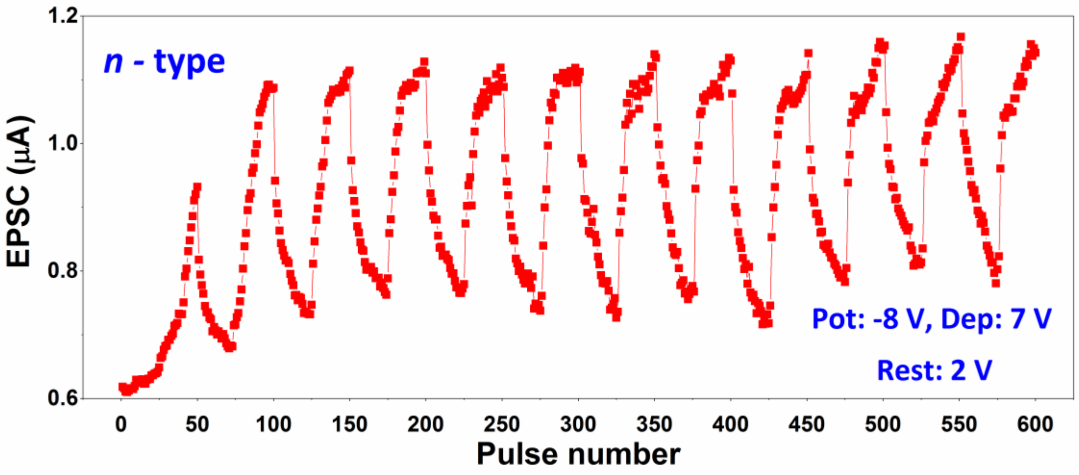

Fig S8 : Multi-cycle repeatability of potentiation-depression characteristics for an *n*-type device. Please note the device takes the initialisation (first) cycle to attain a reliable number of trap states and hence conductance values.

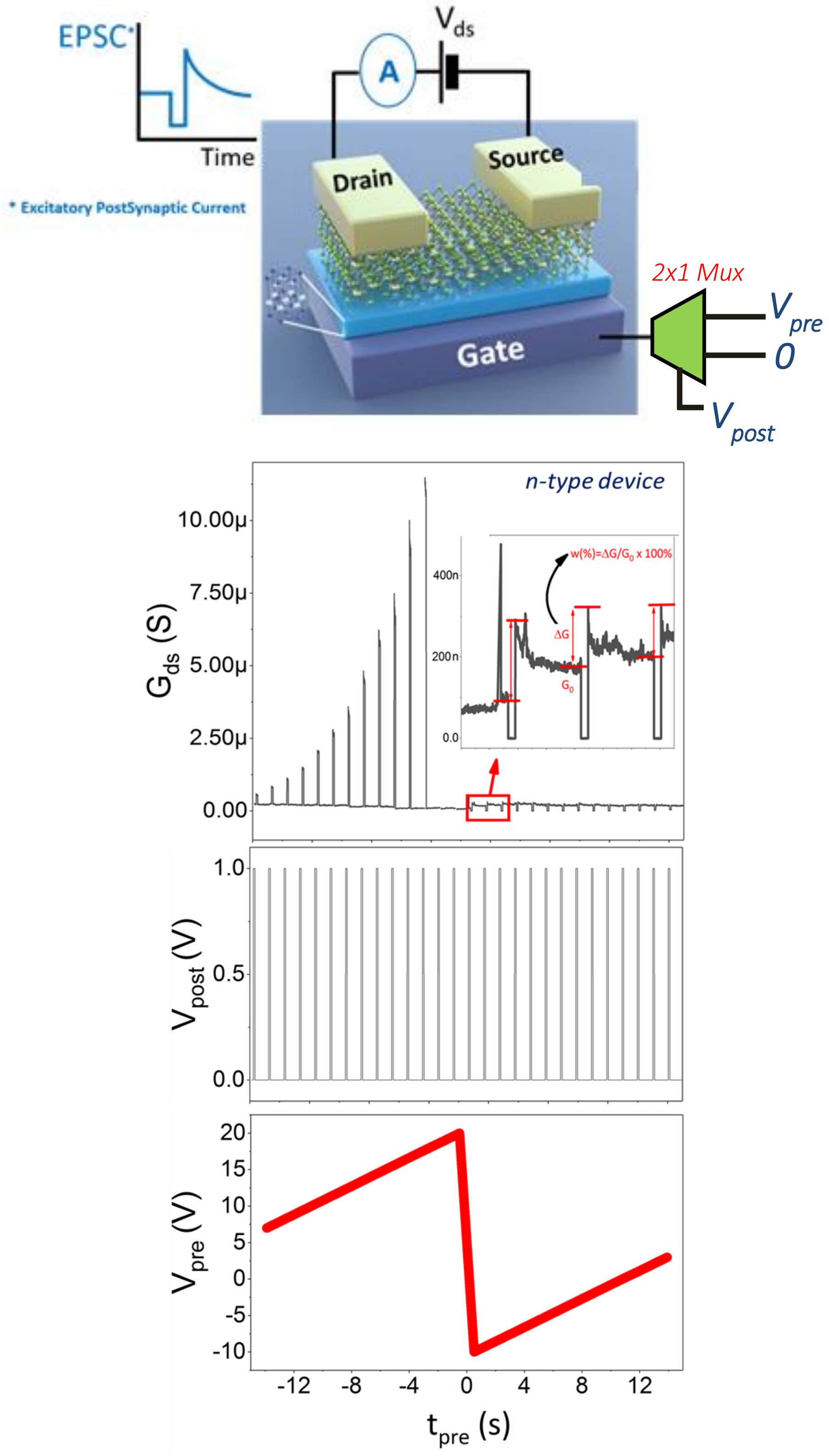

Fig S9. Schematic for spike time dependent plasticity measurements, the change in the conductance of the device from which the synaptic weight change (w%) is calculated in response to the pre and post synaptic pulses. Here, the pre-synaptic Hebbian shaped pulsing scheme facilities the mapping of the time difference (Δt) between the pre- and post-synaptic pulse into the magnitude of applied gate pulses.
